# Ecological Risk Assessment of Amoxicillin, Enrofloxacin, and Neomycin: Are Their Current Levels in the Freshwater Environment Safe?

**DOI:** 10.3390/toxics9080196

**Published:** 2021-08-23

**Authors:** Sangwoo Lee, Cheolmin Kim, Xiaoshan Liu, Saeram Lee, Younglim Kho, Woo-Keun Kim, Pilje Kim, Kyungho Choi

**Affiliations:** 1 Biosystem Research Group, Korea Institute of Toxicology, Daejeon 34114, Korea; sangwoo.lee@kitox.re.kr (S.L.); wookkim@kitox.re.kr (W.-K.K.); 2 School of Public Health, Seoul National University, Seoul 08826, Korea; cjfalswwkd@daum.net (C.K.); liuxiaoshan2006@126.com (X.L.); saeramlee@seoul.go.kr (S.L.); 3 CRI Global Institute of Toxicology, Croen Research Inc., Suwon 16614, Korea; 4 School of Public Health, Guangdong Medical College, Dongguan 511700, China; 5 Nutrition Assessment Team, Seoul Metropolitan Government Research Institute of Public Health and Environment, Gwacheon 13818, Korea; 6 Department of Health, Environment & Safety, Eulji University, Seongnam 13135, Korea; ylkho@eulji.ac.kr; 7 National Institute of Environmental Research, Incheon 22689, Korea; newchem@korea.kr

**Keywords:** veterinary medicine, antibiotics, amoxicillin, enrofloxacin, neomycin, chronic toxicity, risk assessment, surface water

## Abstract

Veterinary pharmaceuticals may cause unexpected adverse effects on non-target aquatic species. While these pharmaceuticals were previously identified as priority compounds in ambient water, their ecological risks are relatively unknown. In this study, a series of chronic toxicity tests were conducted for these pharmaceuticals using algae, two cladocerans, and a fish. After a 21-d exposure to amoxicillin, enrofloxacin, and neomycin, no observed effect concentration (NOEC) for the reproduction of *Daphnia magna* was detected at 27.2, 3.3, and 0.15 mg/L, respectively. For the survival of juvenile *Oryzias latipes* following the 40-d exposure, NOEC was found at 21.8, 3.2, and 0.87 mg/L, respectively. Based on the results of the chronic toxicity tests and those reported in the literature, predicted no-effect concentrations (PNECs) were determined at 0.078, 4.9, and 3.0 µg/L for amoxicillin, enrofloxacin, and neomycin, respectively. Their hazard quotients (HQs) were less than 1 at their average levels of occurrence in ambient freshwater. However, HQs based on the maximum detected levels of amoxicillin and enrofloxacin were determined at 21.2 and 6.1, respectively, suggesting potential ecological risks. As the potential ecological risks of these veterinary pharmaceuticals at heavily contaminated sites cannot be ignored, hotspot delineation and its management are required.

## 1. Introduction

Veterinary pharmaceuticals have been used for the treatment and/or prevention of diseases in both companions and livestock animals. After use, proportions of pharmaceuticals can be excreted from the body unchanged or as active metabolites [1]. In addition, veterinary pharmaceuticals can reach the environment via direct application in aquaculture or through the disposal of the unused [2,3]. Therefore, veterinary pharmaceuticals have been frequently reported in ambient water worldwide [4,5,6,7,8]. Given that pharmaceuticals are designed for specific therapeutic functions, these compounds may cause unexpected physiological effects on non-target species [6,9,10]. Hence, their potential consequences in aquatic environment have been of concern.

Antibiotics and antimicrobials are used to control pathogenic bacteria [4,11] and have been widely used in veterinary medicine to prevent diseases and promote the growth of livestock and fish [12]. In terms of the amount of use, these groups of veterinary pharmaceuticals occupy among the highest ranks in many countries; and hence have been detected in ambient environments at high concentrations, often at as high as µg/L levels [6,10,13]. According to prioritization studies in the United Kingdom (UK) and Korean environments, amoxicillin, enrofloxacin, and neomycin have been suggested as compounds with high hazard potential, mainly due to their higher possibility to reach the environment [2,14]. Because of their potential ecological risks, ecotoxicological assessments have been conducted for many veterinary antibiotics and antimicrobials, but they are often only limited to their acute toxicity [9].

Amoxicillin is a broad-spectrum β-lactam antibiotic that belongs to the penicillin family. Amoxicillin has been used for the treatment of certain gastrointestinal and systemic infections [15]. This compound has been detected at the ng/L level in various countries such as Ghana [16], Turkey [17], Italy [8,18], and Australia [7], with a maximum concentration of 1.65 µg/L. For amoxicillin, no observed effect concentrations (NOECs) were derived at 0.78 µg/L based on its chronic toxicity on blue-green algae [19]. Enrofloxacin is a fluoroquinolone antibiotic that inhibits the activity of bacterial DNA gyrase, which is essential for replication and transcription in prokaryotes [20]. Enrofloxacin has been frequently detected in surface waters worldwide, including Asia [21,22,23,24], Europe [25,26], North America [27], and Oceania [7]. However, ecotoxicity information for enrofloxacin is restricted to acute exposure, and chronic toxicity values are available only for algae and invertebrate species [12]. Neomycin is a water-soluble aminoglycoside that has been used for gastrointestinal infections and mastitis [28]. Nevertheless, both the occurrence and ecotoxicity of neomycin are not very well characterized.

In the present study, the ecological hazards of amoxicillin, enrofloxacin, and neomycin were evaluated using an algae *Pseudokirchneriella subcapitata*, two invertebrate species, *Daphnia magna* and *Moina macrocopa*, and a vertebrate *Oryzias latipes*, representing three trophic levels in freshwater ecosystems. Predicted no effect concentrations (PNECs) for these drugs were derived based on the toxicity information obtained in the present study and those reported in the literature. Potential ecological risks were estimated by comparing the surface water concentrations of these compounds reported in the literature and the PNECs. The results of this study will provide useful information on the potential ecological risks of these veterinary pharmaceuticals and, if necessary, help develop relevant risk management options in freshwater environments.

## 2. Materials and Methods

### 2.1. Test Chemicals

Reagent grade amoxicillin (CAS RN: 26787-78-0, purity ≥ 90%), enrofloxacin (CAS RN: 93106-60-6; purity ≥ 98.0%), and neomycin sulfate (CAS RN: 1405-10-3; 734 μg neomycin/mg) were purchased from Sigma Aldrich (St. Louis, MO, USA). The physicochemical characteristics of the pharmaceuticals are shown in Table S1. Test solutions of each compound were prepared immediately prior to the experiments. The test concentrations for each pharmaceutical that were employed for the acute test were determined by preliminary range-finding tests (data not shown). The concentration range for chronic exposure was determined based on the results of the acute toxicity tests, i.e., the highest exposure concentration of the chronic exposure was set at about one-half to a tenth of an acute EC_50_ of each pharmaceutical. The actual concentrations of the test solutions were measured following the method shown in the Supplementary Materials and Methods, and the average measured concentrations for each pharmaceutical are reported in Table S2.

### 2.2. Test Organisms and Maintenance

All test organisms were maintained at the Environmental Toxicology Laboratory of Seoul National University (Seoul, Korea). *Pseudokirchneriella subcapitata* was cultured in a temperature-controlled shaking chamber at 22 °C, with a shaking speed of 220 rpm [29] under continuous illumination at 4306 lx [30]. The two cladocerans, *D. magna* and *M. macrocopa*, were cultured in-house in M4 media-manufactured following OECD guideline 211 [31]. *Daphnia magna* was maintained at 21 ± 1 °C in 6-L glass jars, and *M. macrocopa* was maintained at 25 ± 1 °C in 3-L glass beakers. *Daphnia magna* and *M. macrocopa* were fed daily with algae. Japanese medaka (*O. latipes*) were cultured in a temperature controlled incubation room (25 ± 1 °C) under a photoperiod of 16: 8 h light:dark. The fish were fed *Artemia* nauplii (<24 h after hatching) twice daily. Water quality parameters such as pH, conductivity, temperature, and dissolved oxygen were routinely monitored.

### 2.3. Toxicity Tests

A 72-h growth inhibition test was carried out for *P. subcapitata*, following the OECD test guideline 201 [29] with a minor modification on the initial cell densities. Three replicates with a cell density of 1.0 × 10^5^ cells/mL were exposed to various concentrations of amoxicillin (0, 1.6, 8.0, 40, 200, or 1000 mg/L), enrofloxacin (0, 1.1, 3.3, 10, or 30 mg/L), or neomycin (0, 0.2, 1.0, 5.0, 25, or 125 mg/L).

For *D. magna* and *M. macrocopa*, 48-h acute tests were performed following the OECD test guideline 202 [32]. Four replicates of five neonates (<24-h old) were exposed to a series of concentrations of amoxicillin (0, 12.3, 37.0, 111, 333, or 1000 mg/L), enrofloxacin (0, 12.5, 25, 50, 100, or 200 mg/L), or neomycin (0, 1.85, 5.55, 16.7, 50.0, or 150 mg/L). The number of immobile organisms was recorded after the 48-h exposure. During the acute tests, the test organisms were not fed.

The chronic 21-d *D. magna* and 7-d *M. macrocopa* tests were conducted following the OECD test guideline 211 [31] and Oh and Choi [33], respectively. Ten replicates with one neonate each (<24-h old) were exposed to various concentrations of amoxicillin (0, 3.70, 11.1, 33.3, 100, or 300 mg/L), enrofloxacin (0, 0.123, 0.370, 1.11, 3.33, or 10.0 mg/L for *D. magna*; 0, 0.247, 0.741, 2.22, 6.67, or 20.0 mg/L for *M. macrocopa*), or neomycin (0, 0.0617, 0.185, 0.556, 1.67, or 5.00 mg/L). The exposure medium was renewed at least three times per week. The mortality of the organisms and the number of living offspring were recorded daily. At the end of the test, the body length of each *D. magna*, from the top of the head capsule to the base of the shell spine, was measured using a stereomicroscope (Dongwon, Bucheon, Korea) as described by Olmstead and LeBlanc [34].

A fish early life stage (ELS) toxicity test was initiated with fertilized eggs (<24 h of spawning) and carried out until 30 d post-hatching (dph) following the OECD guideline 210 [35]. The hatching rate, survival, and growth were measured for exposed or hatched fish. Four replicates (15 newly fertilized eggs per replicate) were exposed to a series of concentrations of amoxicillin (0, 1.23, 3.70, 11.1, 33.3, or 100 mg/L), enrofloxacin (0, 0.005, 0.05, 0.5, 5, or 50 mg/L), and neomycin (0, 0.01, 0.1, 1.0, 10, or 100 mg/L) until 30 dph. At 30 dph, five fish per treatment group were randomly selected and their body length and weight were measured. Fish were anesthetized in ice-cold water following the guidelines of the Seoul National University Institutional Animal Care and Use Committee.

### 2.4. Hazard Quotient Calculation

The hazard quotient (HQ) of each tested pharmaceutical was calculated by dividing the measured environmental concentrations (MECs) reported in the literature by the PNEC derived for each pharmaceutical. Among the available MECs from each location, the maximum values of MEC_mean_ and MEC_max_ were chosen and compared with PNEC for each tested pharmaceutical which was determined following the European Commission [36]. For the calculation of MEC_mean_, concentrations below the LOQ were not included. For the PNEC derivation, toxicity data based on ecologically relevant toxicity endpoints (e.g., mortality, immobilization, reproduction, or growth inhibition) were considered, and the most sensitive ecotoxicological data obtained in the present study and those reported by others were employed. If the HQ value is less than 1, the ecological impact is considered negligible.

### 2.5. Statistical Analysis

The median effective concentration (EC_50_) of the algae was determined using REGTOX ver. 7.0.3 (GNU General Public License, Boston, MA, USA). For the cladocerans, the EC_50_ and associated confidence intervals were calculated by probit analysis using Toxstat^®^ (Ver. 3.5; West, Cheyenne, WY, USA). Fisher’s exact test was used to calculate NOEC for the chronic survival of cladocerans. To analyze the reproduction and growth data of cladocerans and all the data of fish, one-way analysis of variance (ANOVA) and Dunnett’s T post-hoc test were performed using SPSS 20.0 for Windows (SPSS, Chicago, IL, USA). Before conducting the ANOVA, normality of data and homogeneity of variance were confirmed. When necessary, a non-parametric Kruskal–Wallis test was performed. The population growth rate (PGR) for the cladocerans was calculated according to Lotka [37].

## 3. Results and Discussion

### 3.1. Acute and Chronic Toxicity of the Tested Pharmaceuticals

#### 3.1.1. Amoxicillin

All the ecotoxicity data obtained from the current study are summarized in Table 1, along with those reported from previous studies. For algae, the 72-h growth EC_50_ was determined at 213.14 mg/L in the present study. This value was lower than that of González-Pleiter et al. [38] (>1500 mg/L); however, it was much higher than that reported for another algae species (cyanobacteria): For *Synechococcus leopoliensis*, the 96-h EC_50_ was reported at 2.22 µg/L [19]. Compared to green algae, cyanobacteria are generally more sensitive to most antibiotics such as aminoglycosides, macrolides, quinolones, sulfonamides, and tetracyclines [39,40,41]. The difference in sensitivity among algae species might be due to the fact that many antibiotics inhibit protein synthesis of prokaryotic cyanobacteria through binding to ribosome subunits; however, chloroplast division in eukaryotic green algae may not be affected [39].

**Figure 1:**
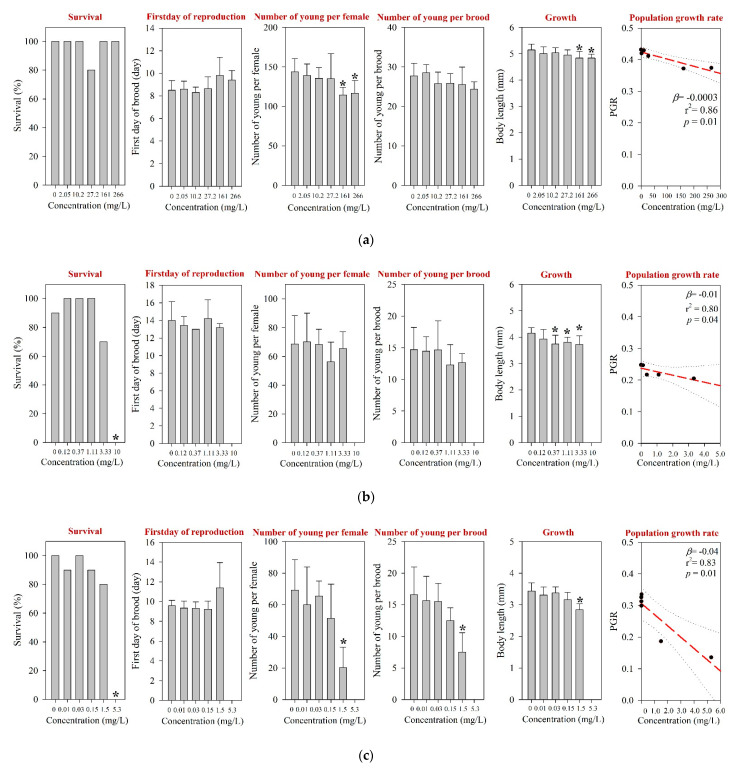
Results of the 21-d chronic *D. magna* test for (**a**) amoxicillin, (**b**) enrofloxacin, and (**c**) neomycin. The results are shown as mean ± standard deviation (*n* = 10). The Asterisk (*) denotes a significant difference in the observation endpoint from that of the control (*p* < 0.05). Monotonous trend was assumed for statistical analysis of the growth of *D. magna* following exposure to enrofloxacin (**b**). Nominal concentration was used for enrofloxacin (**b**). β, slope; r^2^, coefficient of determination; *p*, probability value.

**Figure 2:**
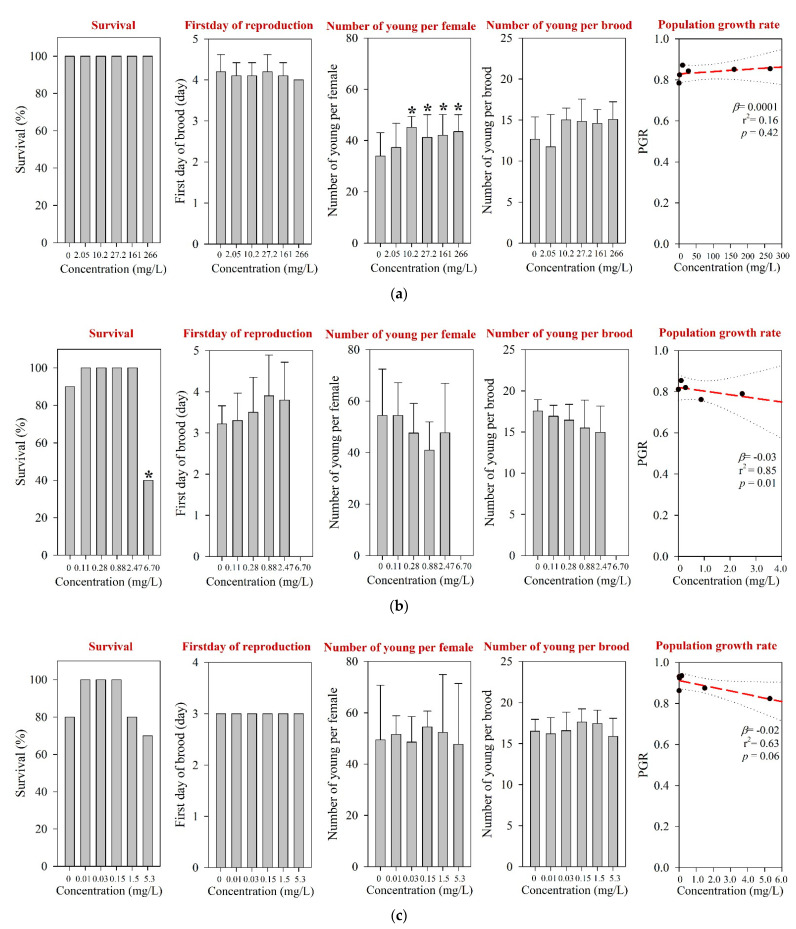
Results of the 7-d chronic *M. macrocopa* test for (**a**) amoxicillin, (**b**) enrofloxacin, and (**c**) neomycin. The results are shown as mean ± standard deviation (*n* = 10). Asterisk (*) denotes a significant difference in the observation endpoint from that of the control (*p* < 0.05). Monotonous trend was assumed as the number of young per female of *M. macrocopa* exposed to amoxicillin (**a**). β, slope; r^2^, coefficient of determination; *p*, probability value.

For *D. magna* and *M. macrocopa*, the 48-h EC_50_s values were determined at >1000 mg/L, which was the maximum experimental concentration (Table 1 and Table S3). These values were comparable to those reported in the literature [12]. The results of the 21-d chronic *D. magna* exposure for the tested pharmaceuticals are shown in Figure 1. The reproduction NOEC of amoxicillin in the 21-d chronic *D. magna* test was determined at 27.2 mg/L (Table 1 and Figure 1a). However, survival and other reproductive-related endpoints, e.g., the first day of reproduction and number of young per brood, were not affected at concentrations up to 266 mg/L. In addition, the population growth rate (PGR) showed a significant decreasing trend (Figure 1a). The results of the chronic *M. macrocopa* exposure for tested pharmaceuticals are depicted in Figure 2. The *M. macrocopa* reproduction NOEC for amoxicillin was determined at 2.05 mg/L, but the change was in a positive direction (Table 1 and Figure 2a). This positive or increasing trend of *M. macrocopa* reproduction by amoxicillin exposure should not be considered beneficial, because, the extent of change was small, and in *D. magna*, we found the opposite direction of the reproduction effect (Figure 1a). For amoxicillin, no chronic toxicity value for cladocerans is available in the literature; therefore, the present data could not be compared.

**Figure 3:**
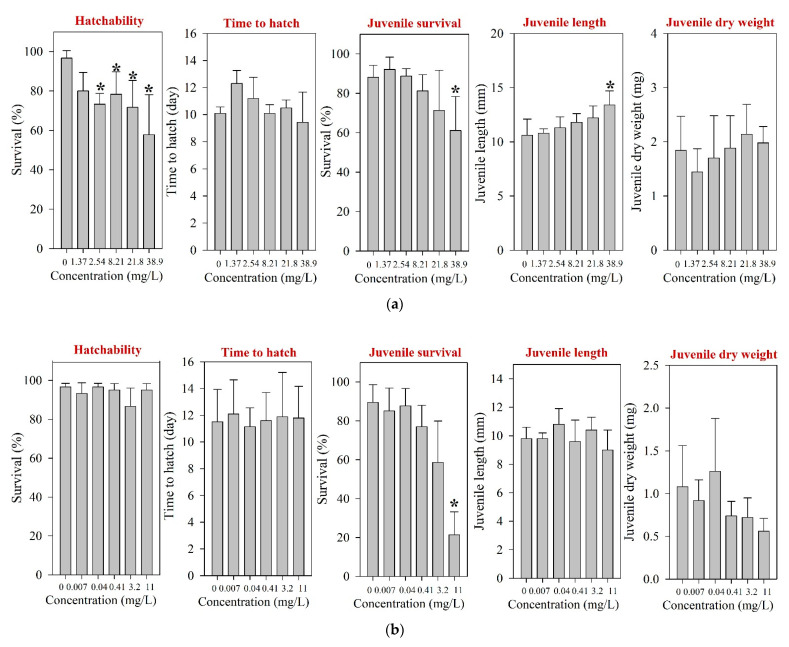

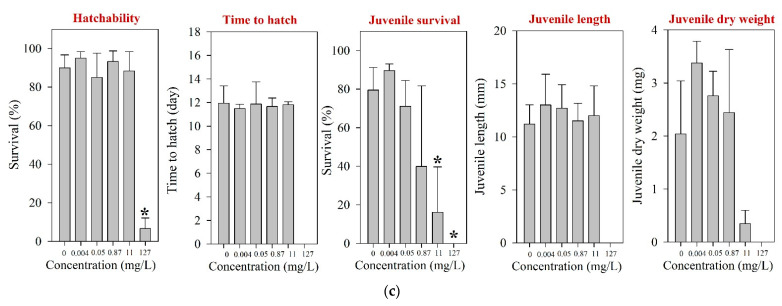
Results of the early life stage test of *O. latipes* for (**a**) amoxicillin, (**b**) enrofloxacin, and (**c**) neomycin. The results are shown as mean ± standard deviation (*n* = 4). Asterisk (*) denotes a significant difference in the observation endpoint from that of the control (*p* < 0.05). Monotonous trend was assumed for statistical analysis of hatchability of *O. latipes* exposed to amoxicillin (**a**).

For fish, only acute toxicity information is available to date [12,42,43]. After 96 h of exposure, LC_50_s were reported at >100 and 1000 mg/L in *Danio rerio* and *O. latipes*, respectively [12,42]. However, for *Tilapia nilotica*, LC_50_ was determined at 0.0357 mg/L [43], suggesting notable variation of sensitivity by fish species. The results of ELS *O. latipes* exposure obtained in the present study for tested pharmaceuticals are shown in Figure 3. After 30 d of ELS exposure of *O. latipes*, we observed the hatchability NOEC at 1.37 mg/L (Figure 3a), and survival LOEC at 38.9 mg/L. Our result shows that the acute to chronic ratio of amoxicillin for *O. latipes* is very high (1000 vs. 1.37 mg/L).

#### 3.1.2. Enrofloxacin

For *P. subcapitata*, the 72-h growth EC_50_ of enrofloxacin was determined at 3.33 mg/L (Table 1). This result corresponded well with previous reports which were made on the same species [20,44]. The EC_50_s values reported for other algal species such as *Chlomydomonas Mexicana*, *Chlorella vulgaris*, *Scenedesmus obliquus*, *Micractinium resseri*, and *Ourococcus mutipsorus* are slightly higher, despite the longer exposure duration [45,46]. The lowest EC_50_ reported for algae species was 0.049 mg/L, which was observed from freshwater cyanobacteria, *Microcystis aeruginosa*, following a 5-d exposure [44].

For *D. magna* and *M. macrocopa*, the 48-h EC_50_s were determined at 20.1 mg/L and 85.2 mg/L, respectively (Table 1 and Table S3). The 48-h EC_50_s from both cladoceran species obtained from the present study are comparable to those reported elsewhere, e.g., for *D. magna* ranging between 15.7 and 56.7 mg/L [12,47,48] and for *M. macrocopa* at 69 mg/L [21]. The EC_50_s values reported for other invertebrates, including *D. curvirostris*, *Gammarus pulex*, and *Physella acuta*, ranged between 4.33 and 133 mg/L [49,50]. Following a 21-d exposure of *D. magna*, survival and growth NOECs were determined at 3.33 mg/L and 0.12 mg/L, respectively (Figure 1b). The body length was significantly reduced at 0.37 mg/L, but reproduction was not affected at concentrations of up to 3.33 mg/L, which was the highest concentration without significant lethal effects. The survival NOEC of *M. macrocopa* was determined at 2.47 mg/L, which was similar to that of *D. magna* (Table 1 and Figure 2b). Due to the low survival rate, the PGRs in both *D. magna* and *M. macrocopa* were significantly reduced in a concentration-dependent manner (Figure 1b and Figure 2b).

Following the fish ELS exposure, survival NOEC was determined at 3.2 mg/L (Table 1), and significant juvenile mortality was observed at 11 mg/L (Figure 3b). However, the hatchability and time-to-hatch of *O. latipes* were not influenced by the exposure at up to 11 mg/L. For fish, ecotoxicity information for enrofloxacin is very limited to date; only one report on acute toxicity is available [12].

#### 3.1.3. Neomycin

For neomycin, the 72-h growth EC_50_ for *P. subcapitata* was determined at 4.60 mg/L (Table 1). This observation is quite different from the reports made on other algae, e.g., *Anacystis nidulans* (6-h NOEC of 0.2 mg/L) and *Microcystis aeruginosa* (24-h NOEC of 0.1 mg/L) [51,52]. Different experimental species and conditions, for example, different cell densities, light intensities, and endpoints were employed in these studies, and hence direct comparison with that of the present study may not be appropriate.

The 48-h EC_50_s for *D. magna* and *M. macrocopa* were determined at 56.0 mg/L and 22.9 mg/L, respectively (Table 1 and Table S3); which were comparable with a previous report [12]. For *D. magna*, chronic survival and reproduction NOECs were determined at 1.5 mg/L and 0.15 mg/L, respectively (Table 1). Neomycin exposure decreased reproduction performance, including the number of young per female and the number of young per brood of *D. magna* (Figure 1c), and PGR. Neomycin exposure led to the steepest decline of the PGR slope for *D. magna* among the three pharmaceuticals tested in this study. Based on the *M. macrocopa* chronic toxicity test, however, no significant changes in both survival and reproduction were observed at all experimental concentrations up to 5.3 mg/L neomycin (Figure 2c), which was above the NOEC reported previously [12]. The PGR of *M. macrocopa* showed a slightly decreasing pattern, with marginal statistical significance (*p* = 0.06).

Following the fish ELS exposure, hatching was significantly affected at 127 mg/L; the hatchability of *O. latipes* at 127 mg/L neomycin was 6.7% (Figure 3c). The survival of juvenile fish was significantly impaired at 11 mg/L neomycin. However, the growth of *O. latipes*, i.e., juvenile length and dry weight, was not altered by the neomycin exposure. Previously, a couple of studies have reported toxicity values of neomycin on aquatic vertebrates, and they were much higher than the survival NOEC (40-d juvenile survival, 0.87 mg/L) of the juvenile fish observed in the present study: A 96-h LC_50_ of 80.8 mg/L was reported for *O. latipes* and an LC_50_ of 2928 mg/L (without specification of the exposure period) was reported for *Anguilla japonica* [12,53].

**Table 1:** Ecotoxicity of tested pharmaceuticals on aquatic organisms obtained from the present study and from the literature.

Pharmaceuticals/ Taxonomic Group	Species	Test Duration /Endpoint	Concentration (mg/L)	Reference
**Amoxicillin**				
Bacteria	*Vibrio fischeri*	5 min, IC_50_	1320.0	Park and Choi [12]
Bacteria	*Vibrio fischeri*	15 min, IC_50_	3597.0	Park and Choi [12]
Algae	*Microcystis aeruginosa*	7 d, EC_50_	0.0037	Lützhøft et al. [40]
Algae	*Microcystis aeruginosa*	7 d, EC_50_	0.00803	Liu et al. [1]
Algae	*Pseudokirchneriella subcapitata*	7 d, NOEC	250	Lützhøft et al. [40]
Algae	*Pseudokirchneriella subcapitata*	72 h, EC_10_	4.75	This study
Algae	*Pseudokirchneriella subcapitata*	72 h, EC_50_	213.14	This study
Algae	*Pseudokirchneriella subcapitata*	72 h, EC_50_	>1500	González-Pleiter et al. [38]
Algae	*Rhodomonas salina*	7 d, EC_50_	3108	Lützhøft et al. [40]
Algae	*Synechococcus leopoliensis*	96 h, EC_50_	0.00222	Andreozzi et al. [19]
Algae	*Synechococcus leopoliensis*	96 h, NOEC	0.00078	Andreozzi et al. [19]
Algae	*Synechococcus leopoliensis*	96 h, LOEC	0.00156	Andreozzi et al. [19]
Aquatic plant	*Lemna gibba*	7 d, EC_10_	>1	Brain et al. [54]
Invertebrate	*Daphnia Magna*	48 h, EC_50_	>1000	Park and Choi [12]
Invertebrate	*Daphnia Magna*	48 h, EC_50_	>1000	This study
Invertebrate	*Daphnia Magna*	21 d, survival NOEC	>266	This study
Invertebrate	*Daphnia Magna*	21 d, reproduction NOEC	27.2	This study
Invertebrate	*Daphnia Magna*	21 d, growth NOEC	27.2	This study
Invertebrate	*Moina macrocopa*	48 h, EC_50_	>1000	Park and Choi [12]
Invertebrate	*Moina macrocopa*	48 h, EC_50_	>1000	This study
Invertebrate	*Moina macrocopa*	7 d, survival NOEC	>266	This study
Invertebrate	*Moina macrocopa*	7 d, reproduction NOEC	2.05	This study
Fish	*Danio rerio*	48 h, EC_50_ premature hatching	132.4	Oliveira et al. [42]
Fish	*Danio rerio*	96 h, LC_50_ embryo, adult	>100	Oliveira et al. [42]
Fish	*Oryzias latipes*	96 h, LC_50_	>1000	Park and Choi [12]
Fish	*Oryzias latipes*	Hatchability NOEC	1.37	This study
Fish	*Oryzias latipes*	Time-to-hatch NOEC	>38.9	This study
Fish	*Oryzias latipes*	40 d, juvenile survival NOEC	21.8	This study
Fish	*Oryzias latipes*	40 d, juvenile growth NOEC	21.8	This study
Fish	*Tilapia nilotica*	96 h, LC_50_	0.03572	Yasser and Nabila [43]
**Enrofloxacin**				
Bacteria	*Vibrio fischeri*	5 min, IC_50_	272.25	Oh [48]
Bacteria	*Vibrio fischeri*	15 min, IC_50_	306.35	Oh [48]
Bacteria	*Vibrio fischeri*	5 min, IC_50_	425.0	Park and Choi [12]
Bacteria	*Vibrio fischeri*	15 min, IC_50_	326.8	Park and Choi [12]
Bacteria	*Vibrio fischeri*	5 min, EC_50_	>8.4	Hernandoet al. [55]
Bacteria	*Vibrio fischeri*	15 min, EC_50_	>8.4	Hernando et al. [55]
Bacteria	*Vibrio fischeri*	30 min, EC_50_	>8.4	Hernando et al. [55]
Algae	*Anabaena flos-aquae*	72 h, EC_50_	0.173	Ebert et al. [20]
Algae	*Chlorella sp.*	72 h, EC_50_	111	Andrieu et al. [21]
Algae	*Chlamydomonas mexicana*	96 h, EC_50_	10.76	Xiong et al. [41]
Algae	*Chlorella vulgaris*	96 h, EC_50_	12.2	Xiong et al. [46]
Algae	*Desmodesmus subspicatus*	72 h, EC_50_	5.568	Ebert et al. [20]
Algae	*Microcystis aeruginosa*	5 d, EC_50_	0.049	Robinson et al. [44]
Algae	*Micractinium resseri*	96 h, EC_50_	12.03	Xiong et al. [46]
Algae	*Ourococcus mutipsorus*	96 h, EC_50_	14.98	Xiong et al. [46]
Algae	*Pseudokirchneriella subcapitata*	72 h, EC_50_	3.1	Robinson et al. [44]
Algae	*Pseudokirchneriella subcapitata*	72 h, EC_10_	0.83	This study
Algae	*Pseudokirchneriella subcapitata*	72 h, EC_50_	3.33	This study
Algae	*Scenedesmus obliquus*	24 h, EC_50_	88.39	Qin et al. [45]
Algae	*Scenedesmus obliquus*	48 h, EC_50_	63.86	Qin et al. [45]
Algae	*Scenedesmus obliquus*	72 h, EC_50_	45.1	Qin et al. [45]
Algae	*Scenedesmus obliquus*	96 h, EC_50_	59.16	Qin et al. [45]
Algae	*Scenedesmus obliquus*	96 h, EC_50_	9.86	Xiong et al. [46]
Aquatic plant	*Lemna minor*	7 d, EC_50_	0.114	Robinson et al. [44]
Aquatic plant	*Lemna minor*	7 d, EC_50_	0.107	Ebert et al. [20]
Aquatic plant	*Myriophyllum spicatum*	14 d, EC_50_	>44.3	Ebert et al. [20]
Invertebrate	*Daphnia curvirostris*	48 h, EC_50_	4.33	Dalla Bona et al. [49]
Invertebrate	*Daphnia magna*	24 h, EC_50_	26.75	Oh [48]
Invertebrate	*Daphnia magna*	48 h, EC_50_	15.7	Oh [48]
Invertebrate	*Daphnia magna*	24 h, EC_50_	131.7	Park and Choi [12]
Invertebrate	*Daphnia magna*	48 h, EC_50_	56.7	Park and Choi [12]
Invertebrate	*Daphnia magna*	48 h, EC_50_ (pH 7.4)	45.8	Kim et al. [47]
Invertebrate	*Daphnia magna*	48 h, EC_50_	16.34	Dalla Bona et al. [49]
Invertebrate	*Daphnia magna*	48 h, EC_50_	20.1	This study
Invertebrate	*Daphnia magna*	21 d, survival, NOEC	5	Park and Choi [12]
Invertebrate	*Daphnia magna*	21 d, reproduction, NOEC	5	Park and Choi [12]
Invertebrate	*Daphnia magna*	21 d, survival, NOEC	3.33	This study
Invertebrate	*Daphnia magna*	21 d, reproduction, NOEC	3.33	This study
Invertebrate	*Daphnia magna*	21 d, growth NOEC	0.12	This study
Invertebrate	*Gammarus pulex*	48 h, EC_50_ (pH7.0)	42.1	Sun et al. [50]
Invertebrate	*Gammarus pulex*	96 h, EC_50_ (pH7.0)	15.6	Sun et al. [50]
Invertebrate	*Moina macrocopa*	24 h, EC_50_	285.7	Park and Choi [12]
Invertebrate	*Moina macrocopa*	48 h, EC_50_	>200	Park and Choi [12]
Invertebrate	*Moina macrocopa*	48 h, EC_50_	69	Andrieu et al. [21]
Invertebrate	*Moina macrocopa*	48 h, EC_50_	85.2	This study
Invertebrate	*Moina macrocopa*	7 d, survival, NOEC	2.47	This study
Invertebrate	*Moina macrocopa*	7 d, reproduction, NOEC	>2.47	This study
Invertebrate	*Physella acuta*	48 h, EC_50_ (pH 7.0)	133	Sun et al. [50]
Invertebrate	*Physella acuta*	96 h, EC_50_ (pH 7.0)	122	Sun et al. [50]
Fish	*Oryzias latipes*	96 h, EC_50_	>100	Park and Choi [12]
Fish	*Oryzias latipes*	48 h, EC_50_	>100	Park and Choi [12]
Fish	*Oryzias latipes*	Hatchability, NOEC	>11	This study
Fish	*Oryzias latipes*	Time-to-hatch, NOEC	>11	This study
Fish	*Oryzias latipes*	40 d, juvenile survival	3.2	This study
Fish	*Oryzias latipes*	40 d, juvenile growth	>3.2	This study
**Neomycin**				
Bacteria	*Vibrio fischeri*	5 min, IC_50_	>1000	Park and Choi [12]
Algae	*Anacystis nidulans*	6 h, NOEC	0.2	Whitton [52]
Algae	*Microcystis aeruginosa*	24 h, NOEC	0.1	Vance [51]
Algae	*Pseudokirchneriella subcapitata*	72 h, EC_10_	4.28	This study
Algae	*Pseudokirchneriella subcapitata*	72 h, EC_50_	4.60	This study
Aquatic plant	*Lemna gibba*	7 d, EC_10_	>1.0	Brain et al. [54]
Invertebrate	*Daphnia magna*	48 h, EC_50_	42.1	Park and Choi [12]
Invertebrate	*Daphnia magna*	48 h, EC_50_	56.0	This study
Invertebrate	*Daphnia magna*	21 d, NOEC	0.03	Park and Choi [12]
Invertebrate	*Daphnia magna*	21 d, survival NOEC	1.5	This study
Invertebrate	*Daphnia magna*	21 d, reproduction NOEC	0.15	This study
Invertebrate	*Daphnia magna*	21 d, growth NOEC	0.15	This study
Invertebrate	*Moina macrocopa*	48 h, EC_50_	34.1	Park and Choi [12]
Invertebrate	*Moina macrocopa*	48 h, EC_50_	22.9	This study
Invertebrate	*Moina macrocopa*	7 d, NOEC	0.5	Park and Choi [12]
Invertebrate	*Moina macrocopa*	7 d, survival NOEC	>5.3	This study
Invertebrate	*Moina macrocopa*	7 d, reproduction NOEC	>5.3	This study
Mollusks	*Crassostrea gigas*	48 h, EC_50_	>800	US EPA, ECOTOX [53]
Fish	*Anguilla japonica*	LC_50_	2829	US EPA, ECOTOX [53]
Fish	*Oryzias latipes*	96 h, LC_50_	80.8	Park and Choi [12]
Fish	*Oryzias latipes*	Hatchability NOEC	11	This study
Fish	*Oryzias latipes*	Time-to-hatch NOEC	>100	This study
Fish	*Oryzias latipes*	40 d, juvenile survival NOEC	0.87	This study
Fish	*Oryzias latipes*	40 d, juvenile growth NOEC	11	This study

EC_50_, median effective concentration; IC_50_, median inhibitory concentration; NOEC, no observed effect concentration; LOEC, lowest observed effect concentration.

#### 3.1.4. Acute to Chronic Ratio

Acute to chronic ratio (ACR) of two cladoceran species which was calculated by dividing the 48-h acute EC_50_ by the chronic NOEC for *D. magna* or *M. macrocopa*, ranged from 34.5 to >487.8 (Table S3). These ACRs are generally within the ranges reported for other pharmaceuticals. In a previous study [56], the mean ACR of aquatic invertebrate for pharmaceuticals was reported at 314 (*n* = 27; range: 1–3108 and median: 17.6). The ACR is useful in ecological risk assessment because a reliable ACR would allow the use of acute toxicity data to estimate chronic effect concentrations [56,57].

### 3.2. Levels of Environmental Occurrence

The tested pharmaceuticals were reported in the aquatic environments worldwide, and these occurrence data are summarized in Table 2. The literature information shows that both amoxicillin and enrofloxacin have been frequently detected in the aquatic environment worldwide, while neomycin has seldom been reported (Table 2). The maximum values of MEC_mean_ reported for amoxicillin, enrofloxacin, and neomycin, in the literature were 0.068 µg/L, 0.087 µg/L, and 1.18 µg/L, respectively (Table 2). It should be noted however that the maximum MEC_mean_ of neomycin was derived from only two countries, India and Korea [58,59,60]. More information is warranted on the environmental occurrences of neomycin in other geographical areas, and this should be a subject of future research. The maximum reported concentrations (MEC_max_) ranged between 1 and 2 µg/L for amoxicillin and neomycin, but enrofloxacin was reported at up to 30 µg/L in the Isakavagu-Nakkavagu rivers of India [13].

### 3.3. PNEC of Each Pharmaceutical

Based on the acute and chronic ecotoxicity information obtained in the present study and in the literature (Table 1), the most sensitive toxicity value that was identified for each compound was 0.00078 mg/L for amoxicillin [19], 0.049 mg/L for enrofloxacin [44], and 0.03 mg/L for neomycin [12]. Because the chronic toxicity data from three representative trophic levels—that is, algae, daphnids, and fish—were available, an uncertainty factor of 10 was used for each of three veterinary pharmaceuticals for the derivation of PNECs [36]. The PNECs that were determined for the tested pharmaceuticals are shown in Table 3, and these are 0.078 µg/L, 4.9 µg/L, and 3.0 µg/L for amoxicillin, enrofloxacin, and neomycin, respectively (Table 3). With an uncertainty factor of 10, the derived PNECs are expected to provide reasonable measures to estimate potential risks of these pharmaceuticals in ambient water. If necessary, however, the PNECs for the tested pharmaceuticals can be further refined with more chronic ecotoxicological data for diverse taxa, and by employing species sensitivity distribution approach.

### 3.4. Ecological Risks

The HQs derived for the MEC_mean_ of amoxicillin and enrofloxacin were less than one, suggesting negligible risks (Table 3), suggesting negligible ecological risks in the aquatic environment in general. However, at MEC_max_, the HQs for amoxicillin and enrofloxacin were 21.2 and 6.1, respectively. This finding implies that both amoxicillin and enrofloxacin can cause potential ecological risks in hotspot areas, e.g., near the sources. Potential risks of both pharmaceuticals especially at the sites with MEC_max_ indicate that efforts for identification of hotspots and development of appropriate risk management may be required for these pharmaceuticals. For neomycin, negligible risks were expected with an HQ of 0.39. However, considering the fact that the occurrence information for neomycin was very restricted, further surveillance is recommended before its ecological risk can be characterized with greater confidence.

## 4. Conclusions

In conclusion, amoxicillin and enrofloxacin were identified as pharmaceuticals of potential ecological concerns in certain hotspot areas. Further efforts are required to identify their sources of contamination, and to investigate the ecological consequences of both pharmaceuticals. For neomycin, environmental monitoring in ambient water should be followed before its ecological risk can be properly characterized.

## Figures and Tables

**Table 2:** Concentrations of amoxicillin, enrofloxacin, and neomycin reported in surface waters worldwide.

Pharmaceuticals /Location	Number of Detect (Total *n*)	LOQ (µg/L)	Concentration (µg/L)			Reference
			Mean	Min.	Max.	
**Amoxicillin**						
**Africa**						
Ghana						
Kumasi region (Rivers)	–(39)	-	-	<LOQ	0.0027	Azanu et al. [16]
**Asia**						
India						
Yamuna River	4 (7)	-	0.18	-	-	Velpandian et al. [60]
Korea						
Four Major River water ^a^	0 (40)	0.00442	<LOQ	<LOQ	<LOQ	NIER [58]
Turkey						
Buyukcekmece Lake	2 (5)	0.0015	0.00291 ^b^	<LOQ	0.00400	Aydin and Talinli [17]
Karasu River	5 (5)	0.0015	0.0214 ^b^	0.00389	0.0639	Aydin and Talinli [17]
Tahtakopru River	4 (5)	0.0015	0.00635 ^b^	<LOQ	0.0142	Aydin and Talinli [17]
Hamza River	4 (5)	0.0015	0.0123 ^b^	<LOQ	0.0573	Aydin and Talinli [17]
Ahlat River	5 (5)	0.0015	0.0406 ^b^	0.00640	1.654	Aydin and Talinli [17]
Beylikcayi River	5 (5)	0.0015	0.0138 ^b^	0.00280	0.0336	Aydin and Talinli [17]
**Europe**						
France						
Seine River	-	0.0392	0.068	-	-	Dinh et al. [61]
Italy						
River Po and Arno	0 (8)	<0.001	<LOQ	<LOQ	<LOQ	Calamari et al. [18]
River Arno (Castelfranco)	4 (4)	<0.00208	0.00557	0.00357	0.00991	Zuccato et al. [8]
River Arno (Limite sull’Arno)	-	<0.00208	0.00377	-	-	Zuccato et al. [8]
River Arno (Pisa)	-	<0.00208	0.00991	-	-	Zuccato et al. [8]
River Po (Monticelli PV)	-	<0.00208	<0.00208	-	-	Zuccato et al. [8]
**Oceania**						
South-East Queensland, drinking water	0 (20)	0.020	<LOQ	<LOQ	<LOQ	Watkinson et al. [7]
South-East Queensland, environmental water	29 (98)	0.020	<LOQ	<LOQ	0.2	Watkinson et al. [7]
**Enrofloxacin**						
**Asia**						
China						
Chentaizi drainage River	3 (4)	0.0001	0.0044	ND	0.0112	Gao et al. [22]
Dagu drainage River	1 (6)	0.0001	0.0002	ND	0.0012	Gao et al. [22]
Duliujian River	2 (2)	0.0001	0.0041	0.002	0.0062	Gao et al. [22]
Guangzhou –Tap water	–(10)	0.00028	0.002 ^b^	ND	0.0083	Yiruhan et al. [62]
Haihe River	4 (9)	0.0001	0.0004	ND	0.001	Gao et al. [22]
Haihe River, tributary	2 (6)	0.0001	0.0012	ND	0.0051	Gao et al. [22]
Huangpu River	2 (38)	0.01134	<LOQ	ND	<LOQ	Jiang et al. [23]
Huangpu River	5 (13)	-	0.0028	ND	0.0146	Chen and Zhou [63]
Nansha River	12 (12)	0.001	0.00867	0.003	0.02	Shao et al. [64]
Qiantang River, Hangzhou	2 (2)	0.027	0.0146	0.0105	0.0187	Tong et al. [65]
River discharging to Laizhou Bay	13 (23)	0.005	0.0106	ND	0.0246	Zhang et al. [66]
River in Shandong province	12 (25)	0.00133	0.00274	0.0002	0.0522	Hanna et al. [67]
Shahu county, Jianghan	19 (20)	0.00145 ^d^	0.02457	0.00017	0.136	Yao et al. [68]
Tai Lake	6 (101)	-	0.00508	-	0.183	Song et al. [24]
Yangtz estuary	4 (28)	0.00168	-	ND	0.00477	Yan et al. [69]
India						
Isakavagu-Nakkavagu Rivers	4 (5)	0.01	0.064 ^b^	ND	30	Fick et al. [13]
Korea						
4 Major Rivers ^a^	5 (40)	0.010	0.0608 ^c^	<LOQ	0.188	NIER [70]
4 Major Rivers ^a^	1 (40)	0.0829	0.0870 ^c^	<LOQ	0.0870	NIER [58]
4 Major Rivers ^a^	8 (80)	0.00316	0.0156 ^c^	<LOQ	0.0300	NIER [59]
4 Major Rivers ^a^	0 (80)	0.0407	<LOQ ^c^	<LOQ	<LOQ	NIER [71]
4 Major Rivers ^a^	0 (80)	0.009	<LOQ ^c^	<LOQ	<LOQ	NIER [72]
4 Major Rivers ^a^	1 (80)	0.008	0.011 ^c^	<LOQ	<LOQ	NIER [73]
Macao						
Macao -Tap water	–(12)	0.00028	0.0040 ^b^	0.0028	0.0052	Yiruhan et al. [62]
Vietnam						
Freshwater near Mekong delta	42 (154)	0.001	0.012 ^b^	< LOQ	0.059	Nguyen DangGiang et al. [74]
Panguasius catfish pond	–(19)	0.02		0.05	0.68	Andrieu et al. [21]
**Europe**						
France						
Seine River	0 (44)	0.01	-	-	< 0.01	Tamtam et al. [26]
Seine River	-	0.011	<LOQ	<LOQ	<LOQ	Dinh et al. [61]
Portugal						
Mondego River	8 (22)	0.025	-	<LOQ	0.1025	Pena et al. [25]
Spain						
Castellon and Valencia provinces	18 (18)	0.009	-	-	0.070	Gracia-Lor et al. [75]
**North America**						
United States						
139 Streams	0 (115)	0.02 ^d^	ND ^b^	-	ND	Kolpin et al. [5]
23 Streams in Iowa, high-flow	0 (23)	0.01 ^d^	ND	-	ND	Kolpin et al. [27]
23 streams in Iowa, normal-flow	0 (23)	0.01 ^d^	ND	-	ND	Kolpin et al. [27]
23 streams in Iowa, low-flow	1 (30)	0.01 ^d^	-	-	0.01	Kolpin et al. [27]
Oceania						
Australia						
South-East Queensland, drinking water	0 (20)	0.001	ND ^b^	<LOQ	<LOQ	Watkinson et al. [7]
South-East Queensland, environmental water	43 (97)	0.001	ND ^b^	-	0.30	Watkinson et al. [7]
**Neomycin**						
**Asia**						
India						
Yamuna River	3 (7)	-	1.18	-	-	Velpandian et al. [60]
Korea						
4 Major Rivers ^a^	1 (40)	0.00008	0.94 ^c^	<LOQ	0.94	NIER [58]
4 Major Rivers ^a^	0 (80)	0.001	<LOQ	<LOQ	<LOQ	NIER [59]

ND, not detected; LOQ, limit of quantification; -, not available. ^a^ Four major rivers in Korea include the Han River, Geum River, Youngsan River, and Nakdong River. ^b^ Median concentration. ^c^ Concentration below LOQ were not included in the calculation of mean values. ^d^ Limit of detection.

**Table 3:** Hazard quotients derived for amoxicillin, enrofloxacin, and neomycin.

Pharmaceuticals	MEC_mean_ (µg/L)	MEC_max_ (µg/L)	Lowest NOEC (mg/L)	AF	PNEC (µg/L)	HQ Based on MEC_mean_	HQ Based on MEC_max_
Amoxicillin	0.068	1.654	0.00078 ^b^	10	0.078	0.87	21.2
Enrofloxacin	0.087	30	0.049 ^c^	10	4.9	0.018	6.1
Neomycin	1.18	1.18 ^a^	0.03 ^d^	10	3.0	0.39	0.39

^a^ The same value as MEC_mean_ was used because MEC_max_ was not available. ^b^ Based on the *Synechococcuse leopoliensis* 96-h growth NOEC [19]. ^c^ Based on the *Microcystis aeruginosa* 5-d growth EC_50_ in the literature [44]. ^d^ Based on the *Daphnia magna* 21-d survival NOEC [12].

## Supplementary Materials

The following are available online at https://www.mdpi.com/article/10.3390/toxics9080196/s1. Table S1: Physicochemical characteristics of tested veterinary pharmaceuticals, Table S2: Nominal and measured concentrations of amoxicillin, enrofloxacin, and neomycin exposure, Table S3: Toxicity value obtained from acute and chronic test of *D. magna* and *M. macrocopa* after acute or chronic exposure to tested pharmaceuticals.
