# Supplementary material for: Ecological Risk Assessment of Amoxicillin, Enrofloxacin, and Neomycin: Are Their Current Levels in the Freshwater Environment Safe?
Source: Toxics. 2021 Aug 23;9(8):196. doi: 10.3390/toxics9080196 (PMC8402334; doi:10.3390/toxics9080196)
Supplement: Supplementary file 1 [file toxics-09-00196-s001.zip › toxics-1284562-SI.pdf]

Article

Ecological Risk Assessment of Amoxicillin, Enrofloxacin, and Neomycin: Are their Current Levels in the Freshwater Environment Safe?

Sangwoo Lee, Cheolmin Kim, Xiaoshan Liu, Saeram Lee, Young-Lim Kho, Woo-Keun Kim, Philje Kim and Kyungho Choi

Supplementary Materials and Methods

Analytical determination of test compounds

The actual concentrations of the test compounds in the exposure media were measured between water renewals using high performance liquid chromatography (HPLC Series 1100, Agilent Technologies, Santa Clara, CA, USA) with triple quadruple mass spectrometry (MS/MS). Operating conditions for analysis were as follows: injection volume 5 μL, flow rate 200 μL/min, model ESI positive gas temperature 400 °C, and capillary voltage 5500 V. The averages of measured concentrations, e.g., before and after the water renewal, were used for presentation of the results and statistical analysis, because some concentrations were not maintained within 20% of differences from the nominal concentration. Measured concentrations for exposure media were summarized in Supplement Table S2.

Table S1. Physicochemical characteristics of tested veterinary pharmaceuticals.

	**Amoxicillin**	**Enrofloxacin**	**Neomycin sulfate (neomycin)**
Structure	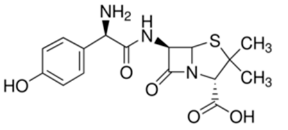	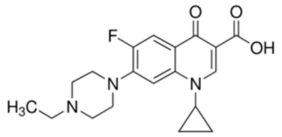	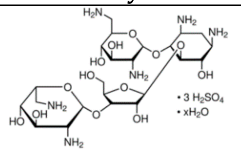
Type	B-lactam antibiotic	Fluoroquinolone antibiotic	Aminoglycoside antibiotic
CAS RN.	26787-78-0	93106-60-6	1405-10-3 (1404-04-2)
Molecular weight	365.4	359.4	908.9 (614.6)
LogK_{OW}	0.87	0.7	(-9.41)
pKa	9.48	7.7	(13.19)

Table S2. Nominal and measured concentrations of the control and treatments that were used for amoxicillin, enrofloxacin, and neomycin exposure.

Pharmaceutical	Type of medium	LOD (μg/L)	Nominal concentration (mg/L)	Measured concentration[a] (mg/L)
Amoxicillin	M4	7.6	0	ND
			3.70	2.05
			11.1	10.2
			33.3	27.2
			100	161
			300	266
	Conditioned Water[b]	7.6	0	ND
			1.23	1.37
			3.70	2.54
			11.1	8.21
			33.3	21.8
			100	38.9
Enrofloxacin	M4	0.2	0	ND
			0.247	0.106
			0.741	0.279
			2.22	0.880
			6.67	2.47
			20.0	6.70
	Conditioned Water[b]	0.2	0	ND
			0.005	0.0066
			0.05	0.043
			0.5	0.41
			5.0	3.2
			50	11
Neomycin	M4	12.4	0	ND
			0.062	0.011
			0.19	0.028
			0.56	0.15
			1.7	1.5
			5.0	5.3
	Conditioned Water[b]	12.4	0	ND
			0.01	0.0044
			0.1	0.053
			1.0	0.87
			10	11
			100	127

ND: Not detected. [a] Average concentration of those measured at the beginning of and after 48 h exposure. [b] The medium for fish (*O. latipes*) toxicity test.

Table S3. Toxicity values obtained from acute and chronic test of *D. magna* and *M. macrocopa* after acute or chronic exposure to tested pharmaceuticals.

Pharmaceuticals	Acute EC_{50} (95% CI)		Chronic NOEC[a]		ACR (this study)[b]	
	D. magna	*M. macrocopa*	*D. magna*	*M. macrocopa*	*D. magna*	*M. macrocopa*
**Amoxicillin**	>1000	>1000	27.2	2.05[c]	>36.8	>487.8
**Enrofloxacin**	20.1 (16.8-23.4)	85.2 (70.4-100.1)	0.12[c]	2.47	167.8	34.5
**Neomycin**	56.0 (39.3-72.8)	22.9 (17.0-28.7)	0.15	>5.3	373.5	NA

Unit in mg/L, EC_{50}: median effective concentration, CI: confidence interval, NOEC: no observed effect concentration, ACR: acute to chronic ratio, NA: not available. [a]The lowest value among the NOECs for survival, reproduction, or growth following 21 d exposure. [b]ACR was calculated from acute EC_{50}/chronic NOEC of this study. [c]NOEC determined based on positive response, i.e., increase in number of neonates per female.
